# A New Integrative Theory of Brain-Body-Ecosystem Medicine: From the Hippocratic Holistic View of Medicine to Our Modern Society

**DOI:** 10.3390/ijerph16173136

**Published:** 2019-08-28

**Authors:** Diego Guidolin, Deanna Anderlini, Guido Maura, Manuela Marcoli, Pietro Cortelli, Giovanna Calandra-Buonaura, Amina S. Woods, Luigi F. Agnati

**Affiliations:** 1 Department of Neuroscience, University of Padova, 35122 Padova, Italy; 2 Centre for Sensorimotor Performance, The University of Queensland, 4072 Brisbane, Australia; 3 Department of Pharmacy and Center of Excellence for Biomedical Research, University of Genova, 16126 Genoa, Italy; 4 Department of Biomedical and NeuroMotor Sciences (DIBINEM), University of Bologna, 40126 Bologna, Italy; 5 IRCCS, Istituto delle Scienze Neurologiche di Bologna, 40139 Bologna, Italy; 6 Structural Biology Unit, National Institutes of Health, National Institute of Drug Abuse-Intramural Research Program, Baltimore, MD 9000, USA; 7 Department of Biomedical Sciences, University of Modena and Reggio Emilia, 41124 Modena, Italy; 8 Department of Neuroscience, Karolinska Institutet, 17177 Stockholm, Sweden

**Keywords:** integrative medicine, ecosystem, heart rate variability, heart-brain interaction, inner speech, depression

## Abstract

Humans are increasingly aware that their fate will depend on the wisdom they apply in interacting with the ecosystem. Its health is defined as the condition in which the ecosystem can deliver and continuously renew its fundamental services. A healthy ecosystem allows optimal interactions between humans and the other biotic/abiotic components, and only in a healthy ecosystem can humans survive and efficiently reproduce. Thus, both the human and ecosystem health should be considered together in view of their interdependence. The present article suggests that this relationship could be considered starting from the Hippocrates (460 BC–370 BC) work “On Airs, Waters, and Places” to derive useful medical and philosophical implications for medicine which is indeed a topic that involves scientific as well as philosophical concepts that implicate a background broader than the human body. The brain-body-ecosystem medicine is proposed as a new more complete approach to safeguarding human health. Epidemiological data demonstrate that exploitation of the environment resulting in ecosystem damage affects human health and in several instances these diseases can be detected by modifications in the heart-brain interactions that can be diagnosed through the analysis of changes in heart rate variability.

## 1. Introduction

Human beings are reaching a deeper awareness that the fate of our species will depend on the wisdom we demonstrate in our interactions with the ecosystem. An ecosystem is defined as a biological community of interacting organisms and their physical environment. As pointed out by Coutts, contemporary ecological models underline human life, health and well-being that are made possible by fundamental ecosystem services [1]. Of essential importance is the water produced by the hydrological cycle, but also the plant and animal materials used as food since humans are heterotroph organisms, hence they can obtain energy only from feeding on other organisms. These services allow humans to exist and to create their survival niches. More than ever, Bateson’s quote should be kept in mind: “The unit of survival is organism plus environment” hence the inclusion of both human and ecosystem health should be essential in view of their interdependence for survivals [2]. Ford and Prescott provide clear definitions about ‘human health’ and ‘ecosystem health’ [3,4]. Thus, as Health Organisation stated in 1948, the human health “is a state of complete physical, mental and social wellbeing and not merely the absence of disease or infirmity”, while the ecosystem health could be defined as a condition in which the ecosystem is able to deliver and continuously renew its fundamental services that allow optimal interactions between humans and the biotic/abiotic components of the ecosystem.

Humans, therefore, can survive and reproduce in optimal conditions only by living in a healthy ecosystem. The spiritual aspect of this relationship has been widely investigated by Soroka et al. [5].

In our opinion it is possible to maintain a healthy co-evolution of the human and ecosystem following some basic concepts that are in operation for both. In particular, the concept of homeostasis, allostasis and the possible formal representations by means of cybernetics of the regulatory mechanisms inside the ecosystem and the human being as well as their interactions can be used.

The main aim of this paper, therefore, is to describe the human being as a “walking ecosystem” (see [6,7] and references cited in these papers), confined within the more general environmental ecosystem with which he has strong reciprocal interactions, leading to a strict interconnection between the human and ecosystem health. Thus, the human health should be described not only as a multidimensional concept covering physical, psychological, and social aspects of wellness but should also take into account that it is strictly interconnected with the ecosystem health which governs the conditions that allow human beings to have access to the fundamental services that are of basic importance for their survival. In this respect, it should also be considered that human beings are a crucial and potentially dangerous component of the environmental ecosystem [8,9].

**Figure 1:**
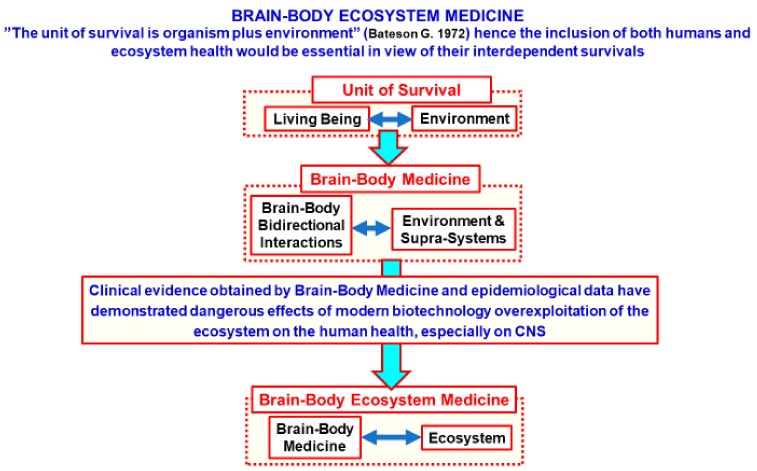
Schematic view of the main steps leading to the brain-body-ecosystem medicine. It should be noted that the first step is the Bateson’s statement about the “unit of survival” and hence, of the interdependence of the living being and the ecosystem in which life is the basic assumption [2]. Another important step in its development has been the brain-body medicine with its focus on the bidirectional interactions between the brain, neuroendocrine organs, and body end-organs such as the heart, the intestine and the kidney. Finally, clinical evidence obtained by the brain-body medicine and epidemiological data have demonstrated the dangerous effects of modern biotechnology overexploitation of the ecosystem on the human health, especially on the Central Nervous System (CNS).

This is the background in which the common perspective of the human and ecosystem health will be proposed as a new integrative view of medicine, namely the brain-body-ecosystem medicine, which is schematically illustrated in Figure 1. It represents an integrative theory, since it combines concepts and central propositions from two or more prior existing theories into a new single set of integrated concepts and propositions. More specifically, the proposed perspective combines the brain-body medicine model (see [10]) with Bateson’s view that the “survival unit is the organism plus the environment” [11] and the Gibbons proposal of the human being as “a walking ecosystem” deeply interfaced to the more general environment [6].

In addition, we will also discuss how the heart-brain bidirectional interplay might suggest crucial hints and preliminary evidence on both the human and ecosystem health. It is hypothesized, in other words, the potential relevance of the heart rate variability as a diagnostic tool that could provide information on the human health conditions in a given environment.

Notably, this new broader view of medicine shows consistency with the ancient view of Hippocrates (460 BC–370 BC) formalized in his fundamental treatise “On Airs, Waters, and Places”.

### 1.1. Brain-Body Medicine: Basic Concepts

In the last two centuries, a basic concept for the study of the living organism was introduced, namely the concept of “homeostasis” that means the tendency of a system, especially of multicellular animals, to trigger a set of coordinated responses from different organs to maintain the constancy of the chemical and physical parameters (essential variables) of the aqueous medium with which its cells interact.

Claude Bernard (1813–1878), the founder of modern experimental physiology, wrote that: “biological systems tend to maintain stability while adjusting to conditions that are optimal for their survival; the stability of the internal environment (the *milieu intérieur*) is the condition for a free and independent life” [12,13]. In the following years, Walter Bradford Cannon (1871–1945) coined the term “Fight or Flight Response” (see below), and expanded Claude Bernard‘s theory introducing the concept of homeostasis [14]. The concept of homeostasis has also been applied in the development of cybernetics and cybernetics concepts (e.g., feedback) which are largely used in physiology and pathology [15,16]. As the constancy of the chemical and physical parameters is loose because their values oscillate around an appropriate set-point, hence there is a dynamic aspect to be considered. On this basis we introduce the concept of “allostasis”. Allostasis are the processes that maintain homeostasis, i.e., stability through suitable changes. As such it is a fundamental aspect of the responses through which organisms actively adjust to both predictable and unpredictable events [17]. The allostatic load refers to the cumulative cost for the body due to allostasis. The allostatic overload can lead to a serious pathophysiological state because the request to face the external and/or internal environment necessities overcomes the organism capability.

In other words, homeostasis describes mechanisms that keep a controlled essential variable constant by sensing its deviation from a “set-point” and feeding back to correct the deviation. Allostasis describes mechanisms that change the controlled variable by predicting the level needed and overriding local feedbacks to meet anticipated demands. Thus, allostasis maintains that the goal of regulation is not constancy but fitness; thus not simply preventing errors and minimizing costs but rather anticipating demands [18,19,20]. Therefore, from a dynamic standpoint, the behavior of a physiological system could be better characterized in terms of attractors, instead of set-points. A dynamic attractor is a set of numerical values of variables towards which the system tends to evolve, for a wide variety of starting conditions. Variable values that get close enough to the attractor values remain close even if slightly disturbed, describing a so-called “basin of attraction” [21,22]. In this respect, the attractor is a very general and unifying concept. A trajectory of the dynamic system in the attractor, indeed, does not have to satisfy any special constraints except for remaining on the attractor, forward in time. It can be a fixed set-point, a finite set of points, a limit cycle, or even a complicated set with a fractal structure as in chaotic dynamical systems [23]. Furthermore, the concept of the attractor is an appealing depiction because it represents a type of stable definable behavior that is very flexible and, in some circumstances, easily altered. The attractor features also have a potential functional meaning, since not only do they mirror the random effects of the inputs on the control system but they are also a consequence of the peculiar characteristics of the control system itself, namely its ability to induce a prompt response of the organism to the environmental challenges.

Summing up: Stimuli from the external environment and/or from the internal medium can modify the value of the controlled variable causing the activation of the sensors connected to the integration center. This center analyses the incoming information and if the controlled variable value is not close enough to the attractor, it sends inputs to the effectors to trigger responses of the opposite sign with respect to the mismatches detected by the sensors (negative feedback control; FB^−^). Keep in mind that allostasis describes the integrated responses of the mechanisms engaged in the change of the controlled variable by predicting the level needed. Those mechanisms are capable of overriding local feedback to meet the anticipated demand. A physiological example is the control of cardiac inter-beat intervals (heart rate, HR) which generates complex chaotic attractors involving a large number of state variables (such as the concentrations of several chemicals in the blood, the metabolic rate, the mental state, the body temperature, and many others) [24]. The FB^−^ control of the HR is a process of paramount importance for every organ, as well as the central nervous system (CNS). The CNS needs an appropriate cardiac output for both its integrative functions and its structural integrity. Mainly HR influences the peculiar features of the brain’s interstitial fluid space (IFS) (hence its internal medium). Our group has proposed that a hierarchic scale can be detected among the various fractions of internal medium in contact with the cells of the different organs of the human body. Moreover, the brain IFS has the highest hierarchic role hence the set-points of the main controlled essential variables of the brain’s IFS are the most carefully controlled [25]. The FB^−^ control of the HR and heart rate variability (HRV) also reflects the integrative actions of the CNS networks involved in the physical and psychic allostasis. There are strict interconnections between these two types of allostasis. Actually, a human being lives in a physical environment, but he also interacts with a socio-cultural environment (i.e., super systems) and continuously creates his own Inner World (see [26,27,28] for a definition) that is peculiar to him, even if it has elements in common with those of other humans with whom he interacts. It is a virtual/mental space in which the subject lives his interactions with the components of the survival unit, namely with his own body and the ecosystem; this interplay has effects on his feeling of well-being, and hence on his psychic homeostasis.

The Inner World, therefore, plays a key role in maintaining a subjective psycho-physical equilibrium [29]. According to Plato, psychic allostasis is characterized by the Eudemonia, an internal peace in which virtue and wisdom converge or, as well stated by Giovanni Reale, a condition of psychic equilibrium in which the subject has reached a deep harmony within his own Inner World [30].

Many of the CNS integrative actions, therefore, are not only finalized for optimal responses of the subject to external environmental changes but are of basic importance for man’s wellbeing because they respond to demands of his Inner World. The CNS integration occurs thanks to the activation of several peripheral apparatuses. This is the basic assumption of the brain-body medicine model that adds a new dimension to research in the fields of mind-body, behavioral, psycho-somatic, and integrative medicine [10]. Brain-body medicine, indeed, focuses on interactions between the brain, neuroendocrine signals and hormones, peripheral pathways and bodily end-organs. Furthermore, interactions between the human being and his microbiota should be considered [31,32,33,34,35].

Bidirectional brain-body pathways can be thought of as the mechanistic substrate that mediates the relationship between psychological and social factors and physical health. Sometimes it is referred to as the mind-body medicine since it focuses on the interactions among the brain, mind, body, and behaviour, and the powerful ways in which emotional, mental, social, spiritual, and behavioural factors can directly affect health. It regards as fundamental an approach that respects and enhances each person’s capacity for self-knowledge and self-care, and it emphasizes techniques that are grounded in this approach.

As previously underlined this theoretical approach is in line with the idea that “Internal Milieu Homeostasis” and “Psychic Homeostasis” (i.e., Eudemonia) are strictly interconnected. This means that the aim of such a therapeutical approach also takes into the account psychotherapy to allow the subject’s eudemonia (hence his psychic homeostasis).The survival value of psychic homeostasis (Eudaimonia) is emphasised by the evidence that it can sometimes override the regulatory mechanisms of bodily homeostasis, i.e., the controls that optimize the human being’s interactions with his external physical environment [19,20,36,37].

In this context it is important to mention Ottaviani’s comments on some implications of the concept of allostasis [26,38,39], which has broadened the investigations of stress reactivity by underlying the paramount importance of assessing not only the subject’s prompt physiological response to stress but also how he usually tries to anticipate possible future stress scenarios as well as how promptly he recovers from the psychophysical costs caused by stress conditions of both biological and psychological origin.

### 1.2. Brain-Body Medicine: on the Important Role of the Bidirectional Heart-Brain Integrative Mechanisms

Psychic homeostasis is characterized by the Eudemonia that from a functional point of view, markedly affects the heart-brain dialogue, since intense emotions, especially if long-lasting, can trigger functionally expensive compensatory mechanisms. Like a cascade process, it leads to an allostatic overload which will result in psychosomatic disturbances, easily detected by alterations in the cardio-vascular control and disturbances in the inner-speech (Pirandello (Luigi Pirandello *Enrico IV* 1922) has described in beautiful poetic sentences that are also from a semeiotic point of view extremely illuminating: “I have sometimes a deep fear even of my blood that pulses in the arteries as, in the silence of the night, gloomy steps in far-located rooms”).

The neurobiological bases of the heart-brain bidirectional interactions have been investigated by Thayer who found evidence of a complex organisation of CNS networks [40]. The complex engaged in integrating different peripheral inputs carrying out the central control of the autonomic nervous system (ANS) in stress conditions, such as the “Fight or Flight Response”.

Stress is an input to the living that elicits a response from the organism that, in most cases, favours survival forcing the organism to adapt to a changed environmental condition. Stress may be acute, chronic, or traumatic. Acute stress is characterized by the recognition of a possible immediate danger and as such activates the fight-or-flight response of the sympathetic nervous system. Chronic stress is characterized by the persistent presence of sources of possible physical danger and/or by psychological stress. Actually, psychological stress can be caused by emotional stress, cognitive stress (e.g., information overload), and perceptual stress (e.g., world view).

According to Thayer’s findings, the Frontal Cortex inhibits the sympathetic nervous system (SNS) but, if this control is reduced, the SNS activity overplays the parasympathetic nervous system (PNS) control. If this is a chronic or a recurring condition, it becomes pathogenic. Thus, when the SNS predominance is associated with reduced Frontal Cortex reactivity, and the subject shows reduced resilience to stress and cardiac symptoms, in particular a reduced HRV. In other words, HRV is an index of the balance between SNS and PNS and it can give us important clues on the psychic conditions of the subject. It should be noted that the latency of SNS and PNS responses are markedly different since sympathetic effects are on the time scale of seconds, whereas the parasympathetic effects are on the time scale of milliseconds, hence within the inter-beat timing of the heart. Thus, the adjustments of the ANS to psycho-physical challenges are mainly due to vagal control [40,41]. On the basis of these evidences the “neurovisceral integration model” (NVI model) has been proposed. It maintains that reduced HR and high HRV are due to the predominance of the vagal control that links the heart and brain through the vagus nerve. In particular, these heart parameters mirror the prefrontal cortex activity and the aspects of the CNS networks that integrate different peripheral inputs carrying out the central control of the ANS in stress conditions [40].

The interplay between the PNS and SNS can be summarized in two conditions:

1. the activity of PNS resulting in the relaxed subject with reduced HR and high HRV
2. the overplay of SNS in anxious or stress situations triggering high HR and low HRV.

In other words, the vagal cardiac tone can be detected by the HRV that mirrors the functional balance of the two components of the ANS that, in turn, results from the integrative actions of the CNS networks involved in cognitive-emotional processes [42,43,44].

It is worth noting that the vagal control is no longer predominant in stress conditions even if the subject is in a resting state. It has also been demonstrated that subjects with higher resting-state HRV had greater flexibility in tasks involving cognitive flexibility [45,46].

These data are of great relevance since they can have important everyday medical implications as:

1. The cardiac activity correlates with the cerebral activity that regulate the psycho-physical allostasis of the subject, hence also basic aspects of his Inner World such as Eudemonia;
2. High, vagally mediated, HRV at rest has been shown to be a reliable index of a higher resilience as evaluated by means of psychometric scales;
3. HR and HRV are easily determinable indices.

So far, we have discussed how the neurovisceral integration model assume as a fact that the cardiac vagal tone, in charge of the HRV mirrors the functional balance of the neural networks implicated in emotion–cognition interactions. A higher HRV may reveal a greater tendency, either in general (i.e., in a resting state) or during a task (i.e., task-related conditions), to rely on the very precise levels of control performed by the prefrontal cortex which are sensitive to goals and context [43]. Thus, the NVI model suggests that HRV should especially be considered as more than just an index of healthy heart function. As a matter of fact, it can also be a reliable index of the capabilities through which the brain integrative actions afford requests of a complex environment. The brain executes a flexible control not only of the heart function but also of several peripheral body parts allowing adaptive regulatory processes [40].

According to the Thayer’s model, the central autonomic network (CAN) has been described as the main CNS network involved in these adaptive regulatory functions [47], which includes the prefrontal cortex (especially anterior cingulate, insula, orbitofrontal, and ventromedial cortex), some limbic structures (mainly amygdala and hypothalamus), and brain stem structures (periaqueductal grey matter, nucleus accumbens, ventrolateral, and ventromedial medulla). The CAN is a complex system whose outputs are integrated into the nucleus of the solitary tract (NTS) and it modulates effectors outside the brain producing adaptive regulatory responses, all this through vagal efferent fibers.

Basically, the CAN output is mediated through the preganglionic sympathetic and parasympathetic neurons that, as far as the cardiac control is concerned, occurs through the stellate ganglia and the vagus nerve, respectively [48].

The brain-body medicine points out how the coupling is bidirectional since the peripheral effectors can lead to changes in the CAN, hence they can have a role in the emotion–cognition interactions.

Indeed, intense emotions, especially if protracted, trigger costly compensatory mechanisms increasing the allostatic loads and often cause dyshomeostasis (an imbalance) even to the point of a breakdown of the psycho-physical equilibrium of the subject. This condition leads to psychosomatic disturbances such as depression, alterations in the cardiovascular control and alterations of the inner-speech. The NVI model has proposed the involvement of the CNS networks with both chemical and physical signals highly activated in these conditions [41,48]. According to the NVI model, several CNS networks engaged in emotional and cognitive functions, especially psychic homeostasis, have the NTS as an important hub. In particular, the brain areas responsible for depressive symptoms are innervated, either directly or indirectly, by projections of afferent vagal fibers terminating in the NTS [40,43]. On the basis of these neuroconnectomics and functional data, researchers have proposed the stimulation of the left vagus nerve (VNS) to treat drug resistant cases of depression [45,49]. The rationale for stimulating the left vagus nerve is that it innervates the atrioventricular node of the heart, therefore it has a smaller effect on the heart rate than the stimulation of the right vagus, which innervates the sinoatrial node.

These findings further underline the relevance of the bidirectional interactions between the heart and brain and the validity of the brain-body medicine. Most importantly, they suggest new therapeutic interventions on the CNS diseases through peripheral VNS.

As a matter of fact, the cardiac activity modulates the integrative actions of the CNS networks involved in cognitive-emotional processes through several, non-mutually exclusive, mechanisms. The main modalities of communication modes between peripheral organs (particularly from the heart and the gut) and the brain are:

1. Vagal afferences: More than 80% of the vagal nerve fibers are afferent fibers;
2. Endocrine signals from the heart (mainly natriuretic peptides) and from peripheral organs (mainly adrenal gland) and from the microbiota (see below brain/gut interactions);
3. Pulsatory waves in the brain parenchyma caused by cerebral artery pulses (see below “The Tide Hypothesis”);
4. The release of micro-vesicles induced by heart beats.

Of interest is that usually, the subject feels that this peculiar condition, either due to environmental stimuli or to internal inputs (even from the inner-speech) can alter his allostatic equilibrium affecting his interactions with the socio-cultural environment in which he is acting.

In regard to pulsatory waves, it was already confirmed in the nineties that emotions able to alter the cardiac function (HR and cardiac output) have an effect on the arterial pulse and, as a result, also alter the pressure waves in the brain parenchyma (Tide Hypothesis) [25,50,51]. In addition to an effect on the volume transmission signal migration, pressure waves interfere with mechanic-sensitive channels [50,51,52,53,54].

Recently, it has been shown that cardiac HR modulates the release of exosomes (a special modality of volume transmission communication) with transient effects on the cell phenotype. This is explained by the fact that exosomes can carry G protein-coupled receptors (GPCRs) and other molecules that, if internalized by the target cells, can give rise to a transient new connectomics in the CNS networks with new integrative abilities in other words to “redeployment” of the CNS networks [55,56].

The relevance of the Tide Hypothesis is indirectly supported by clinical data. For example, it has been shown that the pulsatile reperfusion after cardiac arrest improves the neurologic outcome [57].

## 2. Moving from the Brain-Body Medicine to the Brain-Body-Ecosystem Medicine

The physical and psychic homeostasis depends on the interactions between the brain and all the other organs, where a special role is played by the brain/heart interaction. Such an interaction, however, is in turn significantly influenced by the interactions between the subject and environment.

Living beings, indeed, are elements of the ecosystem and they interact with its other components with the ultimate aim of optimizing their possibilities of survival and reproduction. As discussed before, a basic step of such aim is the allostatic control of essential variables of its IFS that must be maintained within their appropriate set-ranges [14,58]. For humans, the brain IFS allostatic control of the cerebrospinal fluid sodium and glucose concentrations have the highest hierarchic role and ensures an optimal environment for brain cell survival and communication processes in complex cellular networks allowing the CNS integrative functions. Intra-parenchymal pressure waves (caused by the cardiac output) play a role in intercellular communication processes in the brain, favoring volume transmission signal migration and mechano-sensitive ion-channels modulations [25,50,51]. Thus, it is well established that the cerebrospinal fluid sodium and brain IFS pressure waves are two essential variables, hence they are important aspects that point to the relevance of the brain-heart and brain-kidney interactions in the context of the brain-body medicine [59]. Other important aspects of brain-body interactions have been investigated in the last decade, in particular, the fact that vagal afferents to the brain can have psychic effects underlining the importance of body-born signals for psychic allostasis as they can cause or contribute to psycho-pathological conditions. As discussed above, vagal stimulation can have therapeutic effects in major depression [10,60]. The so-called brain-body medicine has therefore received new impetus in the last decades [10,44,61]. The brain-body medicine focuses on complex multiple bidirectional interactions between the brain, neuroendocrine organs, and body end-organs such as the heart, intestine and kidney. These bidirectional brain-body interactions are the functional substrates optimizing the integration of the different and sometimes contrasting needs of psycho-social factors and physical requirements enabling the complex “harmonic state”. A Harmonic state implies the psycho-physical health of man [60].

However, as stated by Bateson “The unit of survival is organism plus environment” and this basic assumption has led us to propose the brain-body-ecosystem medicine. It is evident that the natural selection operates on both the organism and the ecosystem, and evolutionary processes allow more efficient interactions between the components of the unit of survival by means of appropriate changes in some of their features [2]. Robertson and later on Casagrande et al. wrote: “the organisms altered by evolution to adapt to their environment are themselves a significant component of that environment” [62,63]. Therefore both the biotic and abiotic environmental pressure should be considered [64].

In this context, a special aspect of the biotic pressure on humans resides in their own action, since their interactions with the environment are conditioned both by the supra-systems (i.e., the social context) and by the drastic actions that humans perform in the ecosystem [19,59,65]. Those actions are drastic in the sense that they go well above the “creation of survival niches” which is what most species do. In this respect, humans are approaching a point of no-return as a result of their overexploitation of the ecosystem [66]. This has been argued in an intense literary essay and in interesting interview by Konrad Lorenz [67,68]. Human species make high-risk actions capable of upsetting the ecosystem equilibrium between man and nature; hence, humans can be fatal to the “unit of survival” making a new and broader Hippocratic medicine necessary. The new approach will consider the unit of survival in a more comprehensive context by combining the traditional concepts of Hippocrates (see: *On Airs, Waters, and Places*) and the clinical evidence obtained by the brain-body medicine. Epidemiological data on the dangerous effects of modern biotechnology overexploitation of the ecosystem are abundant.

In this context, we will discuss not only some aspects of the relevance of detection procedures to assess the physical and psychological allostasis of human beings, but above all, the interrelations between the ecosystem health and man’s wellbeing. In addition to the complex ecosystem structure, already underlined by Montesquieu, the multifaceted aspects of the social environment have deep effects on human health [69]. We proposed the “Brain-Body-Ecosystem Medicine” theory keeping in mind that the ecosystem in its entirety is “a living organism” [70] (The Gaia hypothesis proposes that living organisms interact with their inorganic surroundings on Earth to form a synergistic and self-regulating, complex system that helps to maintain and perpetuate the conditions for life on the planet [71]). For humans, as for any living organism part of Gaia, the main aims are survival and reproduction. They depend not only on the maintenance of the individual organism allostatic equilibrium but also on the appropriate ecosystem services and on the supra-systems that obviously have a great impact on many aspects of the ecosystem as well as on the human Inner World.

Many years ago, Gould and Vrba, have shown how exaptation can be detected in the evolution of living organisms (in humans too) [72]. This new theoretical perspective has been discussed by Pievani and Serrelli who started from the basic assumption that some physical and/or cognitive features produced by natural selection to perform a function can presently be used for a different function (Such as feathers that might have originally arisen in the context of selection for thermal insulation and not for flying [73]) [74].

Natural selection may subsequently operate upon such a new function to better adapt it to new environmental needs. Exaptation may also have played a role in creating the unique mental abilities that were crucial to the evolutionary success of Homo sapiens and the development of his cultural common background i.e., his complex social organization [75].

Against this background, a new concept has also been advanced: That of “mis-exaptation” defined as a characteristic which, although it may confer positive effects in one field of activity, may reach an inappropriate degree of specialization to the point of having deleterious effects in another field thereby leading to an overall decrease in the fitness of the individual. Processes of this type could be operating in humans as well and could be the basis of some approach they follow towards the environment, causing a decrease of both physical and psychic wellbeing [26].

As an example, let us consider the “inner speech” which usually works as a positive aid to language, learning and reasoning. It can be defined as the subjective experience of language in the absence of overt and audible articulation and is a basic component of self-awareness [66]. As pointed out by Vygotsky (1987) [76], this particular type of talking to oneself has an obvious dialogical quality and, according to this author, inner speech represents the final point in an evolutionary process in which external discourse gradually becomes internalized as a form of verbal thought [77].

## 3. The Hippocratic View of Medicine: The “Brain-Body-Ecosystem Medicine”

Everybody can easily understand that environmental overexploitation and the consequent pollution damaging the ecosystem may have deep effects on human health. Thus, the new holistic view required to a physician is the wellbeing of man ‘in’ his ecosystem, i.e., a “Brain-Body-Ecosystem Medicine”. Today this brain-body-ecosystem medicine holistic view is of increasing importance since man has dramatically altered the ecosystem as it is clear from this short list of some of human’s actions on the ecosystem that likely will cause further disasters [78,79]:

1. Dramatic reduction of biodiversity, such as deforestation, biodiversity loss, and ocean acidification, will impact human health and decrease ecosystem resilience to climate change [79,80];
2. Genetically modified organism production associated to a vertebrate population decline. These ecosystem alterations are accompanied by the impoverishment of “crucial ecosystem services” (e.g., pollination, groundwater) [81,82];
3. Air pollution by micro particles and toxic compounds affecting cognitive capabilities [83,84,85]. A population-based cohort study in Ontario, Canada, where the concentrations of pollutants are among the lowest in the world, has indicated that about 6.1% of dementia cases are due to PM^2.5^ and NO_2_ [86]. Furthermore, air pollution increases depressive symptoms [87]. Air pollution and noise synergistically work in reducing cognitive capabilities [88,89], in altering the blood-brain barrier and the adaptive stress responses [85];
4. Air pollution decreases HRV even in young and healthy subjects and generally has a negative effect on the ANS cardiac control [90,91];
5. “Solastalgia” (Solastalgia is a neologism, invented by the Australian environmental philosopher Glenn Albrecht, to give greater meaning and clarity to environmentally induced distress. Open cut coal mining and the construction of new power stations had transformed this formerly pastoral landscape. Influenced by various environmental thinkers who linked man-made environmental stress leading to “land-sickness” (which, unlike other environmental stresses, did not lead to an environmental recovery) with psychic stress among the population of the particular environment, he developed the concept of solastalgia [92] Their sense of place, their identity, physical and mental health and general wellbeing were all challenged by unwelcome change. Moreover, they felt powerless to influence the outcome of the change process). [93]: A syndrome due to a drastic alteration of the environment where the subject has spent most of his life [94,95,96];
6. Global epidemiological evaluation shows that 3.2% of pathologies in the world are caused by air pollution [97,98].

It is evident that the dangerous man-made alterations of the ecosystem are extremely hazardous and there is an urgent need for humans to take long-term responsibility for their dealings with the ecosystem.

Of particular relevance in this context are the effects of the ecosystem alterations on CNS and ANS. In this respect, it is of interest to consider the example of the “Solastalgia” syndrome, since it allows a better understanding of the links between the ecosystem health and mind health, and the cumulative impact of climatic and environmental changes on mental, emotional, and spiritual health (see [99]. As Louv [100] pointed out, “if climate change occurs at the rate that some scientists believe it will, and if human beings continue to crowd into de-natured cities, then solastalgia will contribute to a quickening spiral of mental illness”. Actually, not only the physical health implications of climatic and environmental changes are well documented [101], but also the emotional, mental, and spiritual health implications (hence the mindset) can be detected [102,103]. Epidemiological data, for instance, demonstrated that the ecosystem deterioration can cause CNS alterations inducing neuropsychiatric diseases such as depression and cognitive disturbances that in turn compromise the rational management of the ecosystem [66]. Altogether these findings raise a further concern since they open the possibility that the psychic disorders caused by the human-made damage to the ecosystem could become part of a positive feedback able to shatter crucial components of the entire “survival unit”. Our group has delineated this point using mathematical models of human-environment interactions [66]. It has been illustrated how human-made damages of the ecosystem can undermine the condition of human health and cause “cognitive deficits” that favor a reduction in the capabilities to foresee future scenarios and to select adequate policies. Ford et al. warned that “*yet the environment and human health remain poorly integrated within research, policy and practice*.” [3].

Thus, the “Brain-Body-Ecosystem Medicine” should be the new integrated approach capable of not only studying the function of the human body but also the environmental conditions that affect the patient’s health. Of course, it should take advantage of the available diagnostic tools and specialized branches of medicine such as public health and hygiene. It is a logical consequence of such a broad and integrated approach that different procedures should be followed according to the different environments in which the patient lives and operates, including his occupational health responsibilities.

Notwithstanding the always more diffuse awareness of the man-made damage to the ecosystem, the deleterious processes are continuously increasing. For example, the overall Earth (global average land and ocean temperature) has warmed by some 0.85 °C between 1880 and 2012 [79].

Thus, it is compulsory for the ecosystem survival that humans retain the capability to forecast future scenarios of their interactions with the ecosystem. Man has to operate to save the integrity of such a complex system that allows life using a rational approach [80]. As a result of our precarious ecosystem equilibrium, therefore, it is of paramount importance that the Hippocratic view of medicine takes into account at least three interconnected components:

1. Ecosystem; 2. Human Psychophysics allostasis; 3. CNS and ANS networks especially the vagal control.

From these three interrelated components it is possible to deduce that man’s health depends on his actions towards the ecosystem. As discussed before, the vagal control is of great importance not only as a crucial connection between the CNS and peripheral body parts, but also for its contribution to the psychic allostasis. The bidirectional connection of the vagus nerve between the brain and body also explains why HRV could be a useful tool to evaluate the conditions of the three components mentioned above. In fact, monitoring the vagal cardiac control by observing the HRV, may allow the evaluation of the efficacy of the cognitive-emotive responses of the subject under examination, as suggested by Hachem and Laborde [104,105]. As a consequence, the vagal cardiac control might also provide measures of the damage caused by the ecosystem deterioration on the psychophysical allostasis of the subject.

**Figure 2:**
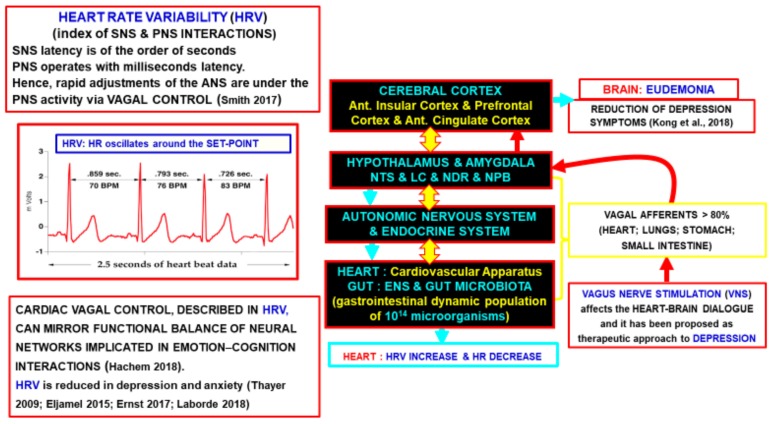
Schematic representation of the crucial role played by the vagal control on the physical and psychic homeostasis of the subject and of the possible diagnostic value of the heart rate variability (HRV). It also indicates the potential therapeutic approach of the vagus nerve stimulation (VNS) for treatment resistant depression.

A schematic view of all these interplays is shown in Figure 2.

### The Hippocratic Approach to Medicine in the Framework of Popper’s Worlds

Based on the idea that health and wellbeing should be analyzed in the context of the ecosystem, we propose to use Popper’s three worlds model of a detailed analysis for man to find the right equilibrium within and among these different but interrelated worlds (W1, W2, and W3) [106].

Let us briefly summarize the main features of these worlds and then examine how man can effectively move inside and between them and maintain his allostatic psycho-physical equilibrium.

**Figure 3:**
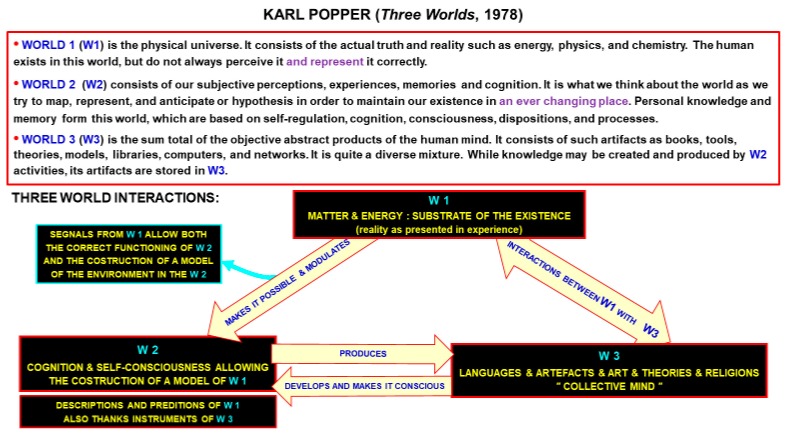
Schematic representation of Popper’s three worlds [106].

Each of these three worlds can be distinguished based on their features (see Figure 3):

World 1 (W1) is the physical world which comprises non-living objects, physical energies and living beings. As underlined by Popper the subdivision between living and non-living objects is not always clear.

World 2 (W2) is the mental world, hence it consists of psychological states or processes, memories and subjective experiences. It is possible to further distinguish the fully conscious experience from dreams, imaginations, or subconscious experience. In other words, World 2 is the mental domain that harbors mental processes, our feelings of pain and pleasure, as well as, our perceptions and observations. It is in this world that we forecast probable future scenarios and we debate with ourselves possible decisions. To a certain extent, this is the world described by Hippocrates when he said that through the brain “*we distinguish the ugly from the beautiful, the bad from the good, the pleasant from the unpleasant*.” Popper’s conclusion is that *human suffering belongs to world 2; and human suffering, especially avoidable suffering, is the central moral problem for all those who can help*. Therefore, it has a crucial relevance for the humanistic approach of the “Hippocratic Physician” to the patient.

World 3 (W3) is the only world uniquely belonging to humans since it consists of typical products of the human mind that are both tangible and non-tangible products. Some intangible products are languages, musical compositions, stories, religions and scientific theories. Tangible objects of W3 include machines and computers that aim to increase the human power on W1. Other tangible objects, that mainly aim to give actual evidence to beauty, are monuments, architectures (e.g., churches), paintings and sculptures. The products of W2 and W3 result from capabilities which are peculiar to human beings and are important actors of his Eudemonia, as discussed in a fascinating book by Young: *Man’s particular genius is for communication by symbols, which is improved by Art and Literature that are major contributors to Human Homeostasis* [107]. We can now understand why the psychic homeostasis in humans has unique characteristics that should be considered by the Hippocratic physician, because it impacts on his Eudemonia. The staying of humans in W1 is made possible and optimized thanks to developmental and learning processes based on genetic and CNS knowledge. The latter plays a crucial role in the upgrading of W2 but also of W3 [68]. Animals and humans are capable of sharing W2 with other members of their species, and have reached W3 achievements, which in some cases are shared by their entire specie. Blakemore concluded that “*the pinnacles of intelligence are exploited by the entire society. In human culture, this has led to the emergence of a kind of communal intellect the Collective Mind of man-that has pushed forward his biological progress at a prodigious rate [75].*”

The three worlds are intermingled, and they contribute to the creation of the Inner World of each individual. We also shouldn’t forget the relevance of the Inner World as the body’s internal landscape which continuously moves across conscious and unconscious levels. Such a landscape is the result of the CNS integrative functions that send inputs to the Inner World. Those inputs are nervous and chemical signals originating from cardiovascular, pulmonary, gastrointestinal, genitourinary, nociceptive, chemosensory, osmotic, thermoregulatory, immune and autonomic systems. The final result is a complex “Interoception” to which the microbiota also contributes [108]. Obviously, interoception is fundamental for the psycho-physical allostasis being involved not only in basic homeostatic physical processes but also in the maintenance of different mental health conditions. As a matter of fact, interoception dyshomeostasis can cause different types of disorders like anxiety, depression, bulimia, anorexia, addiction and somatic symptom (see [109] and references here cited). Making obvious the importance of having a full range of diagnostic indices of the interoceptive signals but such a huge picture of the internal landscape involves several invasive approaches, which tend to elicit physiological perturbations and have a high economical cost. Interesting field of investigations are the non-invasive diagnostic approaches based on the vagal control where the main afferent pathways from the internal organs to the brain are monitored. Laborde tackles this aspect by asking some basic questions that the physician should always keep in mind: *How healthy are individuals? How effective is their thinking, their stress management, their emotion regulation? How effective are they at developing social relationships?* Laborde et al. introduce the “vagal tank theory”, a metaphoric name [105]. They propose the cardiac vagal control as a suitable non-invasive, low cost indicator of the psycho-physiological allostatic control of the subject. It is an indicator of the processes activated to trigger prompt appropriate goal-directed behaviors managing changing circumstances, and to surmising possible future scenarios. Although the vagal control can be a suitable index of the processes that maintain the organism health in a challenging environment, it is not an exhaustive one [110,111]. The cardiac vagal control can alter the HR responding to several interoceptive inputs as well as, environmental requests both from the environment (W1) and from socio-cultural context (W2) and even from W3. Our complex reality shows how the heart influences behavior and how this relationship is reciprocal [112].

Apart from the vagal tank theory, other theories describing the complex links are the polyvagal theory and the neurovisceral integration model [40,41,111,113]. Of particular interest to us is the neurovisceral integration model which postulates that the cardiac vagal control is associated positively to a large range of positive outcomes regarding executive functions, emotion, and health, displaying overall a better self-regulation of the organism [40,111]. Functional evidence and neuroanatomical studies have demonstrated the crucial role played by the vagus nerve. In addition it has been shown that the brain structures involved in self-regulation and those involved in cardiac control largely overlap, specifically structures in the prefrontal cortex [40,111,114]. Vagal afferent fibers are largely scattered through key organs in the human body whereby CNS can obtain an accurate description of the interoceptive landscape through the vagus nerve. This accurate description enables the triggering of the appropriate autonomic, endocrine, and behavioral responses through central reflex pathways especially through the NTS [115]. The interoceptive inputs are integrated to external inputs and on this basis the organism gives the appropriate response with adaptive modulatory feedbacks [116]. As far as the hub role played by the NTS, several brain areas involved in behaviors relevant for depression are innervated, either directly or indirectly, from projections of afferent vagal fibers terminating in the NTS. This is the neurophysiological background on which the VNS is based to treat resistant depression [45] (The VNS procedure involves implantation of a stimulation generator connected to bipolar electrodes that are placed around the left vagus nerve. The rationale for stimulating the left vagus nerve to treat resistant depression (or epilepsy) is that it innervates the atrioventricular node of the heart so as to have less of an effect on HR than the right vagus, which innervates the sinoatrial node. Only patients who had failed four or more antidepressant trials were included. Here, a response rate of 35.7% was found after 12 months). However, as pointed out by Carreno *the mechanisms by which VNS benefits patients nonresponsive to conventional antidepressants is unclear, with further research needed to clarify this* [45].

Last, a peculiar aspect of the ecosystem with which humans interact is the microbiota, i.e., the about 10^14^ microorganisms (50 bacterial phyla and about 100–1000 bacterial species) that colonize his small intestine with a very large exchange surface (about 300 m^2^) making it the widest surface of exchange between humans and the external environment. We can now also define humans as a superorganism that consists of the host and its symbionts making together a “holobiont,” with their collective genome known as “hologenome” [117].

During the last centuries the partner microbiota has undergone marked changes due to the modern alterations in diet, lifestyle and medical care [118]. This means that the brain-body ecosystem medicine must take into account gut microbiota and in particular, the microbiota–gut–brain axis. Given that gut microbiota impacts on various CNS integrative actions, it has a role in the pathophysiology of numerous mental and neurological diseases [119].

In support of this view, it has been shown that the reduction in microbiota diversity occurs in ageing diseases such as depression, Alzheimer’s disease, and Parkinson’s disease [120].

On this basis, new therapeutic approaches have been proposed that are aimed to target the microbiota composition by means of prebiotics, probiotics and even fecal transplantation. Preliminary data indicate that, in some patients, these new therapeutic approaches can improve health [117].

## 4. Conclusions

**Figure 4:**
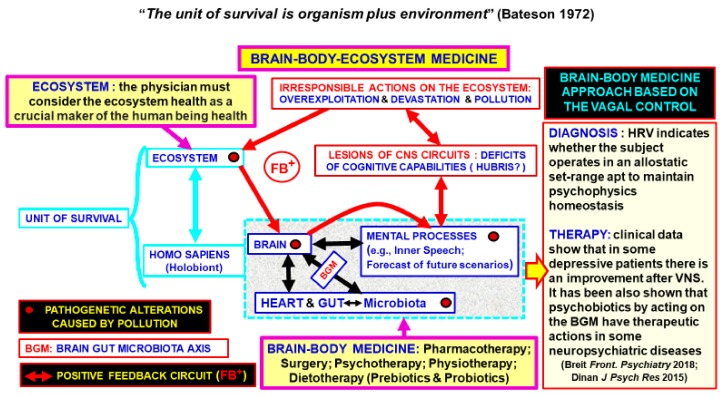
Schematic representation of the basic assumptions of the brain-body-ecosystem medicine model: The physician must consider the ecosystem health as a crucial component of human beings health and the brain-body medicine approach based on the vagal control can be of paramount diagnostic importance to detect disturbances of the physical and psychic homeostasis of the subject.

The present paper proposes a new view of the brain-body-medicine by analyzing the human being as an ecosystem (since he is harboring a microbiota) that interacts with the ecosystem that surrounds him (the environmental ecosystem). Hence, human being health depends on the environmental ecosystem that surrounds him conditioning the brain-body-microbiota interactions. Against this background, the brain-body-ecosystem medicine is proposed where actually two ecosystems are considered namely the human microbiota and the environmental ecosystem (see Figure 4).

It should be noted that the gut-microbiota is a component of the human being multi-facet complex microbiota since the human being is a “walking ecosystem” that interacts with the environmental ecosystem. The physician must consider both ecosystems and their interactions to properly act in order to preserve human being health. For further details see the text and in particular “Conclusions”.

This is an important issue since, especially in the present times and even more in the future, the two ecosystems are rapidly changing due to the actions of the human beings that are contraption-makers and food modifiers. These human capabilities have been very important for the evolutionary success of homo sapiens, but, especially in the last century, they have become dangerous for both the ecosystems, especially for the environmental ecosystem, and as a consequence for human survival [121].

In a previous paper, we have advanced the term “Hubris” to denote the mis-exaptation of human cognitive capabilities, which can result in a presumption to believe in his godlike power over the two ecosystems. Some dangerous effects (especially neurobiological aspects) of the ecosystem damage on human health have been briefly discussed (see also [118,122]).

It is essential to remember that pathogenic changes caused by the overexploitation of the environmental ecosystem and alterations in the microbiota can cause cognitive deficits which could favor Hubris. This in turn, will create a positive FB^-^ between human overexploitation and alterations of the two ecosystems and cognitive deficits with disastrous consequences [66].

In conclusion, there is an urgent need for humans to take a long-term responsibility for their dealings with the two ecosystems, not only because severe physical and mental illnesses are caused by dangerous man-made alterations of both ecosystems, but also because these psychic disorders are likely part of a positive feedback that is able to shatter crucial components of the entire “survival unit”.

Thus, the new approach to medicine should consider the patient as a component of an ecosystem and take into account the Hippocratic view based on both an accurate semeiotic examination and an empathic approach to the patient. Obviously, the approach should combine the most recent advances of biotechnology and a better/cautious utilization of the ecosystem services.

Hippocrates introduced the concept of “crisis” which in medicine is the turning point at which a disease either kills the patient or natural healing processes end up with improvements. Nowadays, the human population is facing an environmental ecosystem crisis as demonstrated by a clear-cut datum namely that the global average surface temperatures is increasing resulting in deleterious changes in the environmental ecosystem (The year 2016 was the warmest since modern recordkeeping began in 1880 and the 3rd year in a row to set a new record for global average surface temperatures. Health is the human face of our climate calamity. In the “Airs, Waters, and Places” series, we intend to explore the direct and indirect health consequences of climate change from many scientific perspectives. In doing so, we hope to bring not only insight but also policy solutions to a problem often contemplated with despair. Even the authoritative report from the 2015 *Lancet* Commission on Health and Climate Change, replete with grim statistics and analyses, found a reason for optimism. “Tackling climate change,” it said, “could be the greatest global health opportunity of the 21st century”). Thus, human beings must acknowledge the ecosystem through careful utilization of resources.

## Abbreviations

ANS	Autonomic Nervous System
CAN	Central Autonomic Network
CNS	Central Nervous System
FB^−^	Negative Feedback
HR	Heart Rate
HRV	Heart Rate Variability
IFS	Interstitial Fluid Space Fluid
NTS	Nucleus of the solitary tract
NVI	Neurovisceral integration
PNS	Parasympathetic Nervous System
SNS	Sympathetic Nervous System
VNS	Vagus Nerve Stimulation
